# The host protein SSR4 mediates PRRSV-induced endoplasmic reticulum stress via interaction with Nsp2

**DOI:** 10.1128/jvi.00266-26

**Published:** 2026-04-23

**Authors:** Yingchao Li, Hongyan Gao, Zhong Liu, Man Lu, Yang Shen, Yajing Xing, Yu Wang, Xiaotong Wu, Pingping Yang, Hongjie Yuan, Yanmeng Hou, Yumei Cai, Baoquan Li, Yihong Xiao

**Affiliations:** 1 Department of Fundamental Veterinary Medicine, College of Veterinary Medicine, Shandong Agricultural University 34734, Tai’an, Shandong, China; University of Michigan Medical School, Ann Arbor, Michigan, USA

**Keywords:** PRRSV, Nsp2, ER stress, SSR4, antiviral drugs

## Abstract

Porcine reproductive and respiratory syndrome (PRRS) is a major economic burden to the global swine industry. Here, we identify the endoplasmic reticulum (ER) translocon component SSR4 as a critical host factor co-opted by PRRSV. We demonstrate that the viral non-structural protein Nsp2 physically interacts with SSR4 via its PLP2 and hypervariable domains and selectively upregulates its expression during infection by prolonging its protein half-life. Functional studies revealed that SSR4 is a proviral factor essential for efficient PRRSV replication. Mechanistically, SSR4 is required for the full activation of the PRRSV-induced ER stress response, specifically modulating the PERK-eIF2α and IRE1α-XBP1 axes of the unfolded protein response. Notably, Nsp2 itself acts as a key inducer of ER stress and mediates the upregulation of SSR4, suggesting a potential feed-forward loop that sustains a virus-favorable ER environment. This relationship is finely balanced, as pharmacological disruption of ER homeostasis using either the inducer tunicamycin (TU) or the chemical chaperone 4-phenylbutyric acid (4-PBA) potently inhibited viral replication. Importantly, TU and another inducer, dithiothreitol, exhibited potent, broad-spectrum antiviral activity against multiple PRRSV genotypes in both cell lines and primary porcine alveolar macrophages. Our study delineates a novel pathogenesis model where PRRSV Nsp2 hijacks SSR4 to engineer a proviral ER stress niche. The Nsp2-SSR4-ER stress axis represents a promising target for the development of broad-spectrum antiviral strategies against PRRS.

**IMPORTANCE:**

This study provides significant insights into porcine reproductive and respiratory syndrome virus (PRRSV) pathogenesis by identifying a novel and specific virus-host interface. We demonstrate that PRRSV, through its Nsp2 protein, hijacks a specific component of the host endoplasmic reticulum (ER) translocon SSR4 to orchestrate a tailored ER stress response conducive to viral replication. This mechanism is distinct from a general disruption of the TRAP complex, highlighting a precise viral strategy. Furthermore, the finding that pharmacological agents, which dysregulate this hijacked pathway—particularly ER stress inducers—act as potent, broad-spectrum antivirals challenges the conventional view of ER stress as a uniformly host-protective response. Our work not only uncovers a key molecular determinant of PRRSV replication but also validates the Nsp2-SSR4-ER stress axis as a promising and novel target for the development of much-needed, broad-spectrum therapeutic interventions against this economically devastating swine pathogen.

## INTRODUCTION

Porcine reproductive and respiratory syndrome (PRRS), caused by the PRRS virus (PRRSV), is a highly contagious disease characterized by reproductive failure in sows and respiratory distress in pigs of all ages, resulting in substantial and sustained economic losses to the global swine industry (1). PRRSV is an enveloped, single-stranded positive-sense RNA virus classified within the family Arteriviridae (2, 3). The viral genome encodes both structural and non-structural proteins (Nsps), which are essential for viral replication and play pivotal roles in virus-host interactions. Among these, non-structural protein 2 (Nsp2) is a multifunctional protein that exhibits papain-like cysteine protease activity and is involved in polyprotein processing (4). Moreover, accumulating evidence indicates that Nsp2 interacts with a variety of host proteins to modulate key host cellular processes, including innate immune responses, autophagy, endoplasmic reticulum (ER) stress, and apoptosis, thereby establishing Nsp2 as a major contributor to PRRSV pathogenesis (5–7). The endoplasmic reticulum (ER) is a central organelle responsible for protein synthesis, folding, modification, and transport in eukaryotic cells and is essential for the maintenance of cellular proteostasis (8–10). When the protein-folding demand exceeds ER capacity, the accumulation of unfolded or misfolded proteins triggers ER stress, leading to the activation of the unfolded protein response (UPR). The UPR is mediated by three key ER-transmembrane sensors: protein kinase R-like ER kinase (PERK), activating transcription factor 6 (ATF6), and inositol-requiring enzyme 1 (IRE1) (11–13). This signaling cascade acts to attenuate protein synthesis, enhance ER folding capacity, and promote the clearance of misfolded proteins (14). Many viruses have been shown to exploit or modulate ER stress pathways to establish a cellular environment conducive to viral replication and assembly (15). PRRSV does not merely trigger a passive UPR but actively reprograms it (15–19). For example, the viral glycoprotein GP2a mediates degradation of the molecular chaperone GRP78, thereby augmenting UPR activation (20). In parallel, the non-structural protein nsp2/3 recruits the transcription factor ATF4 to the viral replication complex, directly facilitating viral RNA synthesis. Through such sophisticated manipulation, PRRSV subverts cellular defense mechanisms to support viral replication—a critical aspect of its pathogenicity (15). Nevertheless, the detailed molecular mechanisms through which PRRSV regulates ER stress remain incompletely elucidated.

Signal sequence receptor subunit delta (SSR4, also named SSRD) is a core component of the translocon-associated protein (TRAP) complex (21, 22). Localized to the endoplasmic reticulum membrane, the TRAP complex facilitates the translocation of nascent polypeptides into the ER lumen during translation, thereby playing an essential role in protein secretion and membrane integration (23, 24). Our previous data suggested a potential interaction between SSR4 and PRRSV Nsp2 (25). In this study, we investigated the role of SSR4 in PRRSV-induced ER stress and uncovered a novel virus-host interaction mechanism mediated by the Nsp2-SSR4 axis. These findings provide a molecular basis for the development of new antiviral strategies against PRRS.

## RESULTS

### Nsp2 interacts with SSR4

**Fig 1:**
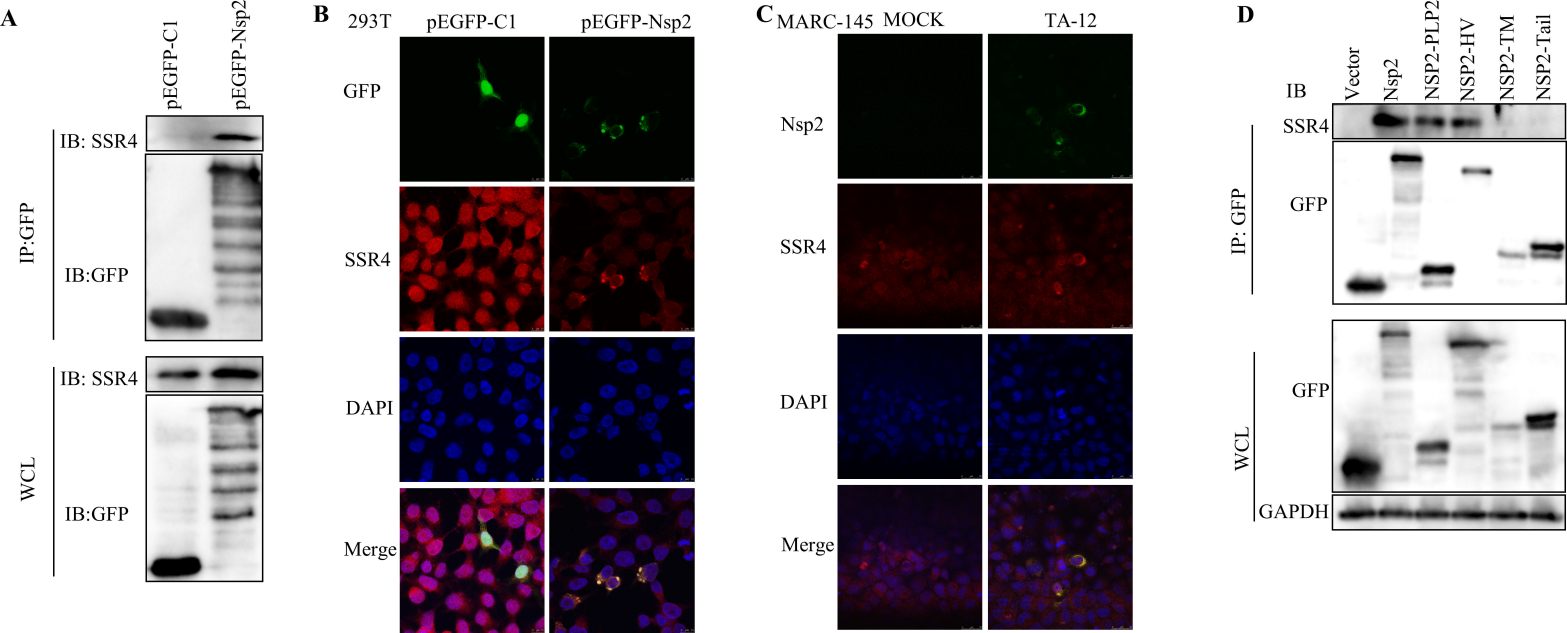
Interaction between Nsp2 and SSR4. (**A**) Co-immunoprecipitation of the Nsp2-SSR4 interaction. Lysates from HEK293T cells transfected with pEGFP-Nsp2 or an empty vector control were subjected to immunoprecipitation with an anti-GFP antibody, followed by Western blot with an anti-SSR4 or anti-GFP antibody. (**B**) Colocalization of ectopically expressed Nsp2 with endogenous SSR4. HEK293T cells transfected with pEGFP-Nsp2 were immunostained with an anti-SSR4 antibody. (**C**) Colocalization of viral Nsp2 with SSR4 during infection. MARC-145 cells were mock-infected or infected with HP-PRRSV strain TA-12 (MOI = 0.1) for 24 h, followed by immunostaining with anti-Nsp2 or anti-SSR4 antibody. In panels B and C, nuclei were counterstained with DAPI. Colocalization appears yellow in the merged images. Scale bars, 10 µm. (**D**) Co-immunoprecipitation analysis of SSR4 with Nsp2 truncation mutants. HEK293T cells were transfected with plasmids expressing full-length GFP-tagged Nsp2 or GFP-fused Nsp2 truncation mutants. Cell lysates were immunoprecipitated using an anti-GFP antibody, and the precipitates were immunoblotted with an anti-SSR4 or anti-GFP antibody. GAPDH served as internal controls for Western blot.

Our previous proteomic analysis identified SSR4 as a putative Nsp2-interacting partner (25). To validate this interaction, we performed GFP-trap immunoprecipitation assays using GFP-Nsp2 as bait. SSR4 was specifically co-precipitated with GFP-Nsp2, confirming their physical association (Fig. 1A). Furthermore, we observed clear co-localization between Nsp2 and endogenous SSR4 in both pEGFP-Nsp2-transfected 293T cells and PRRSV-infected MARC-145 cells (Fig. 1B and C). Domain-mapping experiments further indicated that the papain-like protease domain (PLP2) and the hypervariable region (HV) of Nsp2 are responsible for the interaction with SSR4 (Fig. 1D). Collectively, these data establish that Nsp2 directly interacts with SSR4, suggesting that SSR4 may serve as a critical host factor facilitating Nsp2-dependent processes during PRRSV infection.

### SSR4 is required for efficient PRRSV infection

**Fig 2:**
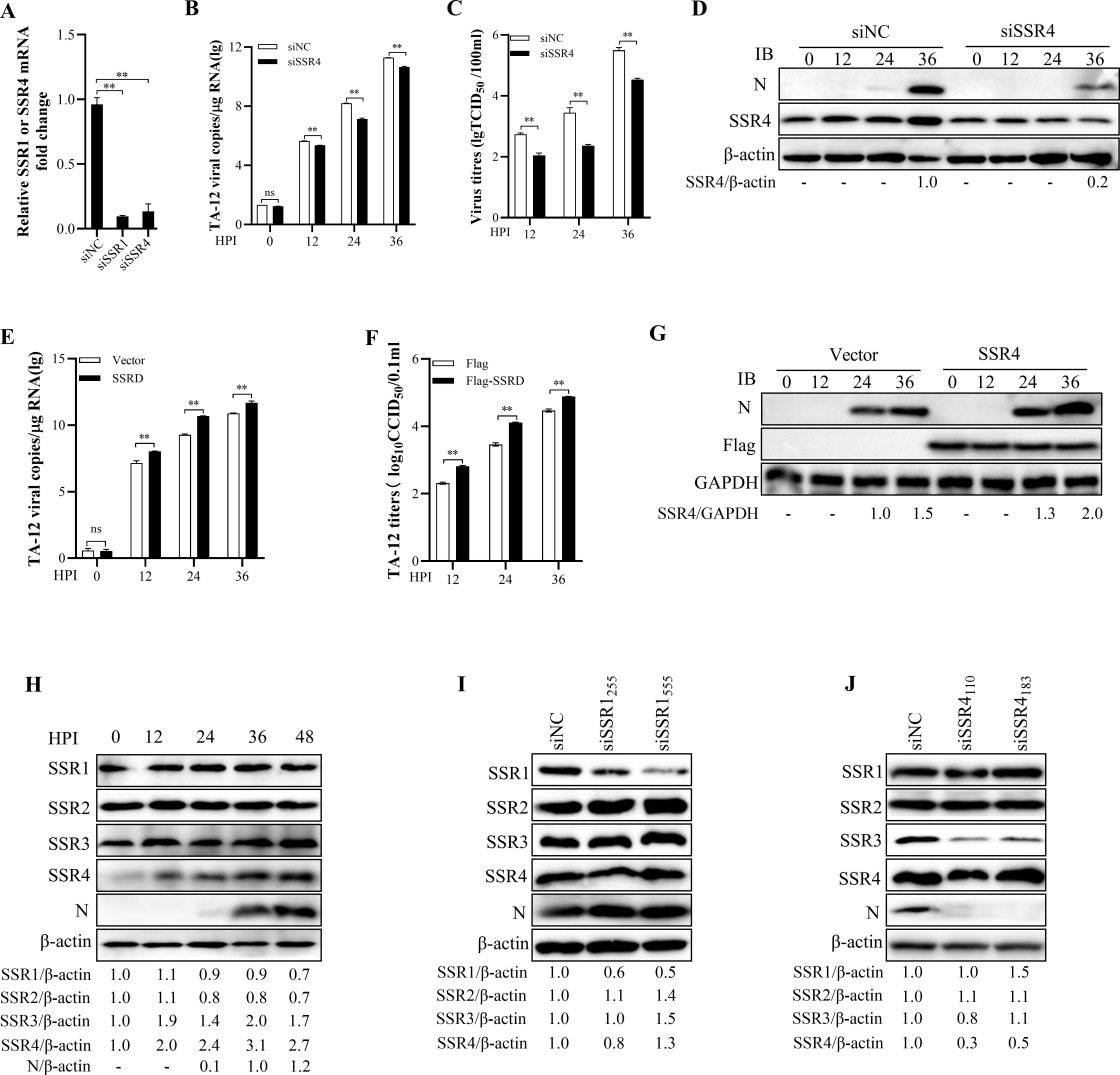
SSR4 facilitates PRRSV infection. (**A–D**) SSR4 knockdown inhibits PRRSV replication. MARC-145 cells were transfected with SSR4-specific siRNA or a negative control (NC) siRNA and subsequently infected with the HP-PRRSV strain TA-12. Cells and supernatants were collected at the indicated hours post-infection (HPI). (**A**) SSR1, SSR4 mRNA levels and viral N gene (**B**) were quantified by RT-qPCR. (**C**) Viral titers in supernatants were determined by the TCID₅₀ assay. (**D**) Viral proteins (N) and SSR4 were analyzed by Western blot. (**E–G**) SSR4 overexpression promotes PRRSV replication. MARC-145 cells were transfected with an empty vector or a Flag-tagged SSR4 expression plasmid, followed by infection with strain TA-12. Viral N gene mRNA levels were measured by RT-qPCR (**E**). Viral titers were determined by TCID₅₀ assay (**F**). Protein expression was analyzed by Western blot (**G**). (**H–J**) PRRSV infection selectively upregulates SSR4. MARC-145 cells infected with TA-12 were harvested at the indicated time points (**H**), and protein expression of the TRAP complex subunits (SSR1-4) was analyzed by Western blot (**I and J**). GAPDH and/or β-actin served as internal controls for qRT-PCR and Western blot. Data are presented as the mean ± SD from three independent experiments, each performed with independent experimental repeats. Statistical significance was determined by an unpaired two-tailed Student’s *t*-test (*P < 0.05, **P < 0.01; ns, not significant).

To assess the functional relevance of the Nsp2–SSR4 interaction for viral infection, we knocked down SSR4 expression using siRNA and evaluated PRRSV replication. Silencing of SSR4 significantly impaired the infection efficiency of the TA-12 strain, as indicated by reduced viral protein expression and RNA levels (Fig. 2A through D). To confirm this finding, we overexpressed SSR4 in MARC-145 cells. Overexpression of SSR4 enhanced PRRSV protein and RNA levels (Fig. 2E and F) and increased viral titer (Fig. 2G). These results demonstrate that SSR4 functions as a proviral factor during PRRSV replication.

Since SSR4 is a component of the multi-subunit TRAP complex, which consists of SSR1, SSR2, SSR3, and SSR4, we further investigated the specific contribution of SSR4 to PRRSV replication. Our data showed that only SSR4 expression was upregulated upon infection, whereas the expression levels of other TRAP complex subunits remained unchanged (Fig. 2H). To examine whether the proviral role of SSR4 extends to other TRAP components, we knocked down SSR1 and assessed its effect on PRRSV infection. Depletion of SSR1 did not significantly affect viral infection (Fig. 2I and J), indicating that the proviral function is specific to SSR4. Moreover, knockdown of neither SSR4 nor SSR1 substantially altered the expression of other TRAP complex subunits, suggesting that the observed effects are not due to disruption of the complex’s structural integrity. Together, these findings indicate that PRRSV infection selectively upregulates SSR4 expression without markedly affecting other TRAP components, supporting a model in which the virus specifically modulates SSR4 to promote its own replication.

### SSR4 modulates in PRRSV-induced ER stress

**Fig 3:**
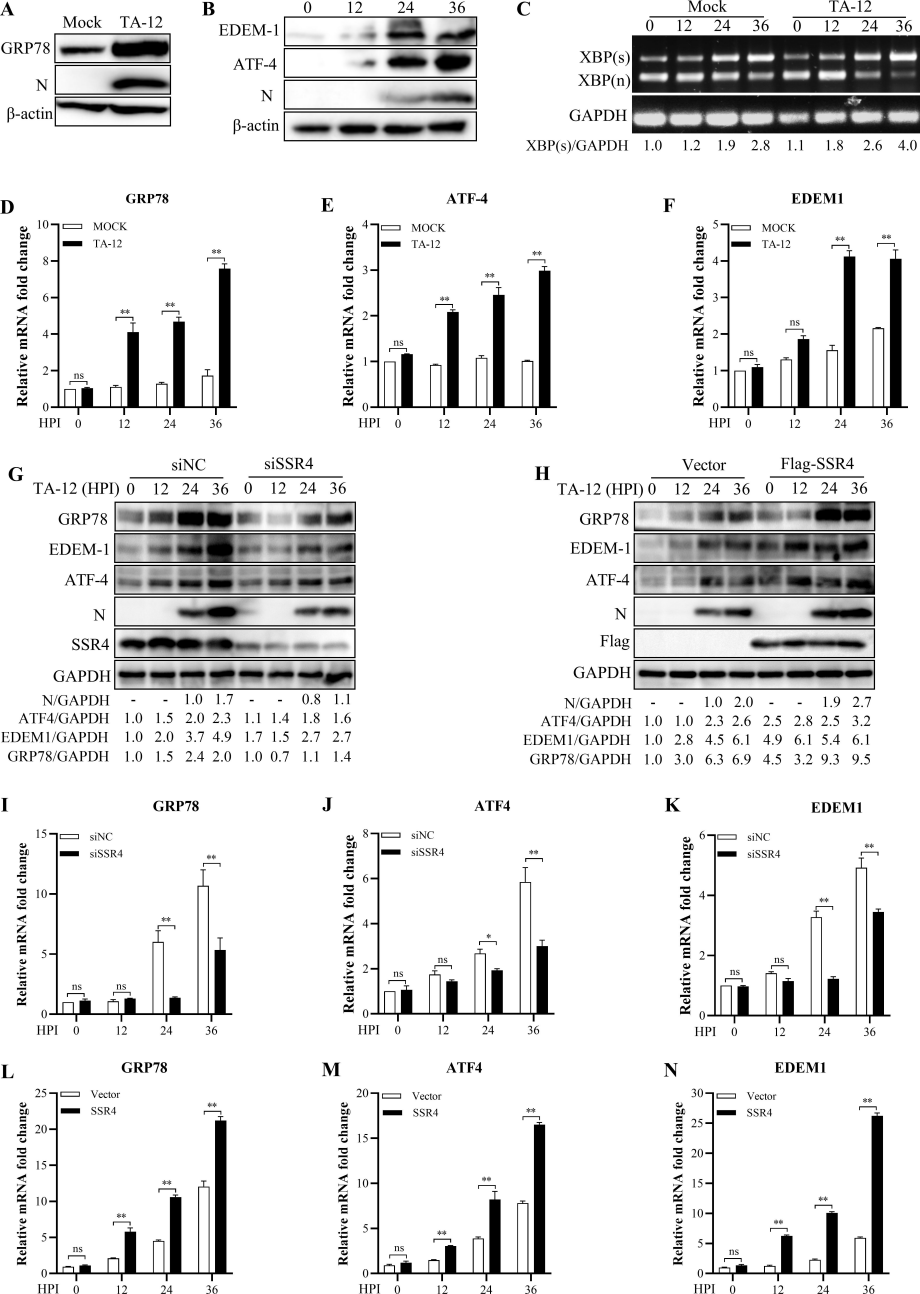
SSR4 is essential for the activation of the PRRSV-induced ER stress response. (**A–G**) PRRSV infection activates the ER stress response. MARC-145 cells were infected with the HP-PRRSV strain TA-12, and the cellular proteins and the mRNAs were prepared at 0, 12, 24, and 36 HPI. (**A**) Protein levels of GRP78 were assessed by Western blot at 36 HPI. (**B**) Time-dependent induction of EDEM1, ATF4, and N protein levels was analyzed by Western blot. (**C**) Splicing of XBP1 mRNA was analyzed by RT-PCR and PstI digestion at the indicated time points to detect the spliced (XBP1(s)) and unspliced (XBP1) isoforms. (**D-F**) Transcript levels of GRP78 (**D**), EDEM1 (**E**), and ATF4 (**F**) were quantified by qRT-PCR. (**G, I-K**) SSR4 knockdown attenuates ER stress marker expression in MARC-145 cells. MARC-145 cells were transfected with SSR4-specific siRNA (siSSR4) or negative-control siRNA (siNC), followed by infection with strain TA-12. Cells were harvested at 0, 12, 24, and 36 HPI. Protein levels of GRP78, EDEM1, ATF4, and viral proteins were analyzed by Western blot (**G**). Gene levels of GRP78 (**I**), ATF4 (**J**), and EDEM1 (**K**) were quantified by qRT-PCR. (H, L–N) SSR4 overexpression enhances ER stress marker expression. MARC-145 cells were transfected with an empty vector or a Flag-tagged SSR4 expression plasmid and infected with strain TA-12. Protein levels of GRP78, EDEM1, and ATF4 were assessed by Western blot (**H**). Gene levels of GRP78 (**L**), ATF4 (**M**), and EDEM1 (**N**) were measured by qRT-PCR at the indicated time points. In all panels, GAPDH and β-actin served as internal controls for qRT-PCR and Western blot, respectively. Data are presented as the mean ± SD from three independent experiments. Statistical significance was determined by an unpaired two-tailed Student’s *t*-test (*P < 0.05, **P < 0.01; ns, not significant).

Given the established role of the TRAP complex in endoplasmic reticulum (ER) stress responses and the known impact of PRRSV on ER homeostasis (7), we investigated whether SSR4 is involved in PRRSV-induced ER stress. We first confirmed that PRRSV infection activates the ER stress response, as indicated by increased protein levels of the ER chaperone GRP78 (Fig. 3A), the ER-associated degradation mediator EDEM1 (Fig. 3B), and the spliced isoform of XBP1 (XBP1(s)) (Fig. 3C). Transcriptional analysis further showed upregulated expression of GRP78 and its downstream targets ATF4 and EDEM1 in infected cells compared with mock-infected controls (Fig. 3D through F). Knockdown of SSR4 significantly reduced both protein and mRNA levels of the ER stress markers GRP78, EDEM1, and ATF4 (Fig. 3G, I through K). Conversely, overexpression of SSR4 increased the expression of GRP78, EDEM1, and ATF4 at both the protein (Fig. 3H) and transcript (Fig. 3L through N) levels. Together, these results demonstrate that SSR4 is required for the full activation of the ER stress response triggered by PRRSV.

### SSR4 modulates the PERK-eIF2α and IRE1α-XBP1 axes of the UPR during PRRSV replication

**Fig 4:**
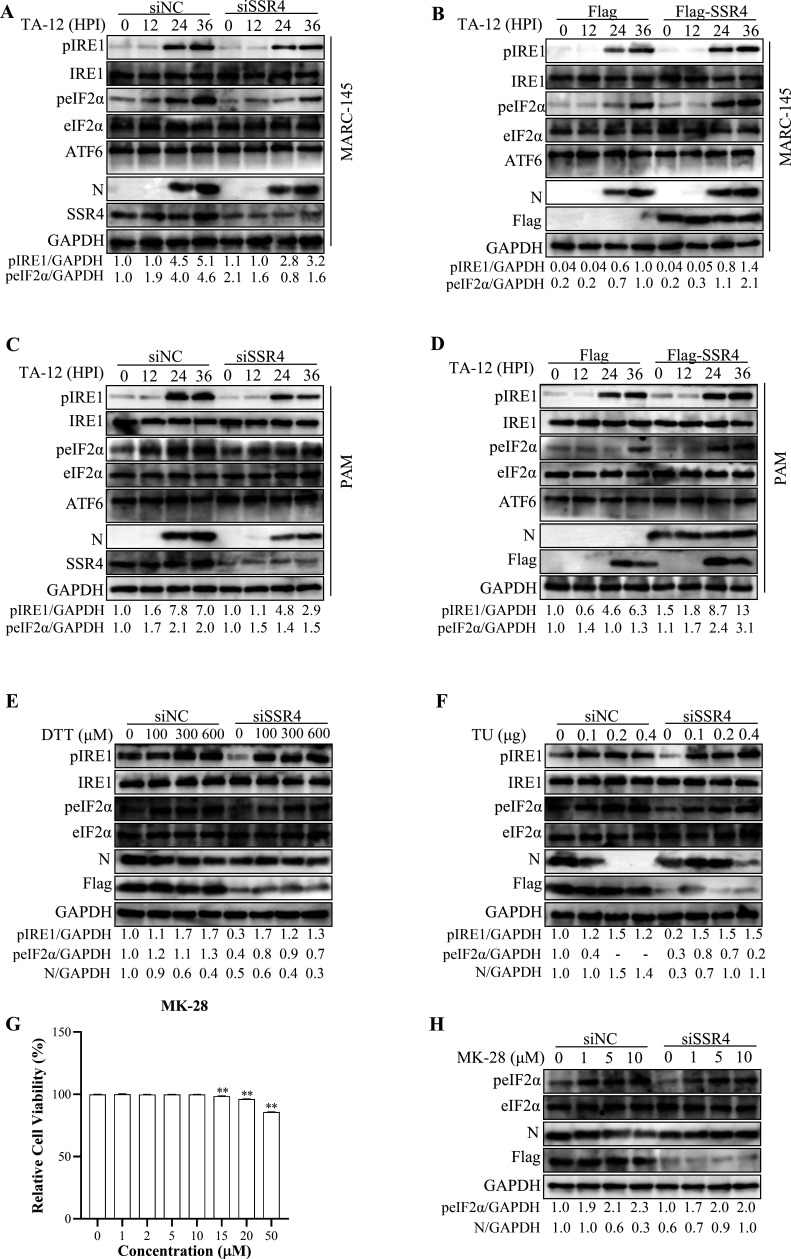
SSR4 modulates the PERK-eIF2α and IRE1α-XBP1 axes in PRRSV-induced UPR. (**A and B**) Role of SSR4 in PRRSV-induced UPR MARC-145 Cells. Cells were transfected with siNC or siSSR4 to knock down SSR4 (**A**) or with an empty vector or Flag-tagged SSR4 expression plasmid to overexpress SSR4 (**B**) for 24 h, followed by infection with PRRSV strain TA-12. Whole-cell lysates were harvested at 0, 12, 24, and 36 HPI and analyzed by immunoblotting. (**C and D**) Role of SSR4 in PRRSV-induced UPR in PAMs. PAMs were subjected to SSR4 knockdown (**C**) or overexpression (**D**), as described for panels A and B, infected with TA-12, and harvested at the indicated time points. Protein levels of UPR markers and viral N protein were assessed by immunoblotting. (**E, F, H**) Pharmacological activation of the UPR rescues the inhibitory effect of SSR4 knockdown. MARC-145 cells were transfected with siSSR4 for 24 h, infected with TA-12, and then treated with increasing concentrations of the ER stress activators dithiothreitol (DTT) (**E**), tunicamycin (TU) (**F**), or the PERK-specific activator MK-28 (**H**). Cells were harvested at 24 HPI, and protein levels of p-IRE1α, p-eIF2α, and viral N protein were analyzed by immunoblotting. GAPDH served as a loading control. (**G**) Cytotoxicity assessment of MK-28 in MARC-145 cells. Cells seeded in 96-well plates were treated with the indicated concentrations of MK-28. Cell viability was measured at 48 h post-treatment using the CCK-8 assay. Absorbance at 450 nm was normalized to untreated control cells (set as 100%). Data are presented as mean ± SD from three independent experiments. Data are presented as the mean ± SD from three independent experiments. Statistical significance was determined by an unpaired two-tailed Student’s *t*-test (*P < 0.05, **P < 0.01).

To determine the role of SSR4 in regulating the unfolded protein response (UPR) during PRRSV infection, we examined the activation status of the three canonical UPR branches—PERK, IRE1α, and ATF6—at 0, 12, 24, and 36 h post-infection (HPI). SSR4 overexpression significantly increased the levels of phosphorylated eIF2α (p-eIF2α), a key downstream effector of the PERK pathway, whereas SSR4 knockdown markedly reduced p-eIF2α levels (Fig. 4A and B). Activation of the IRE1α branch was assessed by monitoring IRE1α autophosphorylation (p-IRE1α). SSR4 overexpression elevated p-IRE1α levels, while SSR4 knockdown diminished them (Fig. 4A and B), indicating that SSR4 potentiates IRE1α-mediated signaling. In contrast, no cleaved ATF6 was detected upon either SSR4 overexpression or knockdown, suggesting that the ATF6 branch is not functionally engaged during PRRSV replication under these conditions. Consistent results were obtained in primary porcine alveolar macrophages (PAMs) following SSR4 overexpression or knockdown (Fig. 4C and D).

To further validate the specific role of SSR4 in PRRSV-induced UPR activation, we performed rescue experiments. Knockdown of SSR4 was followed by treatment with the ER stress inducers DTT or TU. Both compounds restored p-eIF2α and p-IRE1α levels in a dose-dependent manner (Fig. 4E and F). Given the prominent role of the PERK-eIF2α axis, we additionally treated SSR4-knockdown cells with MK-28, a specific PERK pathway activator. MK-28 treatment rescued p-eIF2α levels, confirming that SSR4 functions upstream of PERK activation (Fig. 4G). Collectively, these results demonstrate that SSR4 selectively modulates the PERK-eIF2α and IRE1α-XBP1 axes of the UPR during PRRSV infection, while the ATF6 branch remains largely unaffected.

### Nsp2 activates ER stress and upregulates SSR4 expression

**Fig 5:**
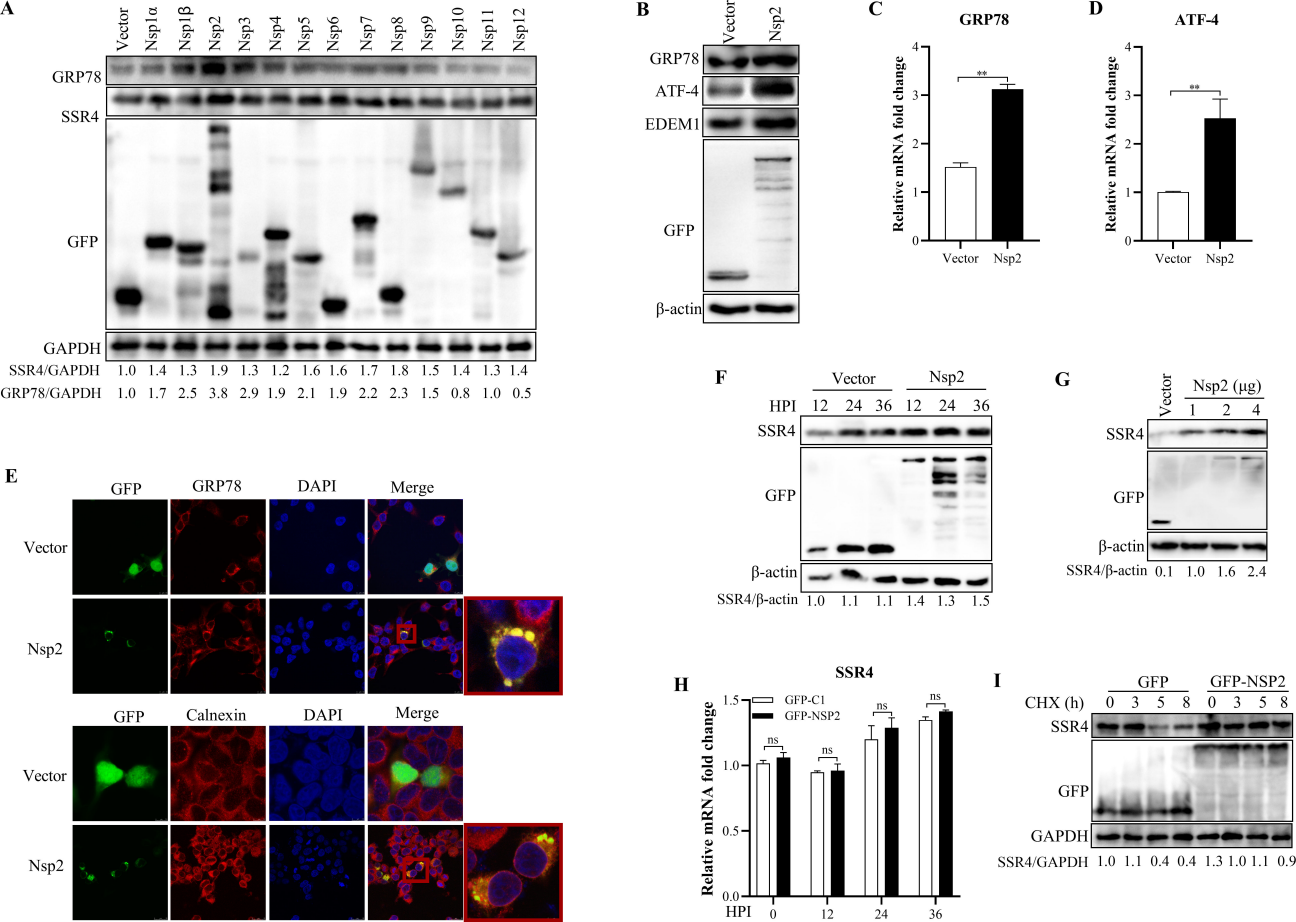
PRRSV Nsp2 activates ER stress and upregulates SSR4 expression. (**A**) Identification of Nsp2 as the major viral inducer of SSR4. MARC-145 cells were transfected with plasmids expressing individual PRRSV proteins. Cell lysates were collected 24 HPI and analyzed by Western blot for SSR4 and GRP78 with specific antibodies. (**B–D**) Nsp2 triggers ER stress activation. MARC-145 cells transfected with pEGFP-Nsp2 or an empty vector were analyzed 24 HPI. (**B**) Protein levels of ER stress markers (GRP78, EDEM1, ATF4) were assessed by Western blot. (**C and D**) Transcript levels of GRP78 (**C**) and ATF4 (**D**) were quantified by qRT-PCR. (**E**) Nsp2 localizes to the endoplasmic reticulum. HEK293T cells expressing GFP-Nsp2 (green) were immunostained with antibodies against the ER markers GRP78 or calnexin (red). Nuclei were stained with DAPI (blue). Scale bars, 10 µm. (**F and G**) Nsp2 upregulates SSR4 expression in a time- and dose-dependent manner. (**F**) HEK293T cells expressing GFP-Nsp2 were harvested at the indicated time points, and SSR4 protein levels were analyzed. (**G**) HEK293T cells were transfected with increasing amounts of pEGFP-Nsp2 plasmid, and SSR4 expression was assessed 24 HPI. (**H**) Nsp2 does not alter SSR4 mRNA levels. MARC-145 cells were transfected with pEGFP-Nsp2 and harvested at 0, 12, 24, and 36 h post-transfection. Total RNA was extracted, and SSR4 mRNA levels were quantified by qRT-PCR. (**I**) Nsp2 enhances SSR4 protein stability. MARC-145 cells were transfected with pEGFP-Nsp2. At 24 h post-transfection, cells were treated with cycloheximide (CHX, 5 μM) to block *de novo* protein synthesis. Whole-cell lysates were harvested at 0, 3, 5, and 8 h post-CHX treatment, and SSR4 protein levels were analyzed by immunoblotting. GAPDH or β-actin served as internal controls for qRT-PCR and Western blot, respectively. Data are presented as the mean ± SD from three independent experiments each performed with separate infections. Statistical significance was determined by an unpaired two-tailed Student’s *t*-test (*P < 0.05, **P < 0.01).

As shown in Fig. 2E, SSR4 expression was upregulated during PRRSV replication. To identify the viral protein(s) responsible for this upregulation, we transfected 293T cells with individual viral proteins and detected endogenous SSR4 expression. Among all viral proteins tested, Nsp2 most significantly increased SSR4 expression (Fig. 5A). Given that Nsp2 was confirmed as an interacting partner of SSR4 (Fig. 1), we further examined its role in ER stress. Ectopic expression of Nsp2 markedly elevated the protein levels of ER stress markers GRP78, EDEM1, and ATF4 (Fig. 5B). Transcriptional analysis confirmed corresponding upregulation of GRP78 and its downstream effector ATF4 (Fig. 5C and D). Immunofluorescence staining demonstrated co-localization of Nsp2 with the ER markers GRP78 and calnexin (Fig. 5E), consistent with its localization to the ER membrane. Notably, Nsp2 expression dose-dependently increased SSR4 protein levels (Fig. 5F and G), establishing a direct link between Nsp2-mediated ER stress induction and SSR4 upregulation. Mechanistically, Nsp2 did not increase SSR4 mRNA levels (Fig. 5H) but, more strikingly, prolonged the half-life of SSR4 protein (Fig. 5I), indicating that Nsp2 promotes SSR4 accumulation primarily through post-translational stabilization. These findings position Nsp2 as a key viral factor that coordinates ER stress activation and SSR4 induction during PRRSV infection.

### Pharmacological targeting of ER stress exhibits broad-spectrum anti-PRRSV activity

**Fig 6:**
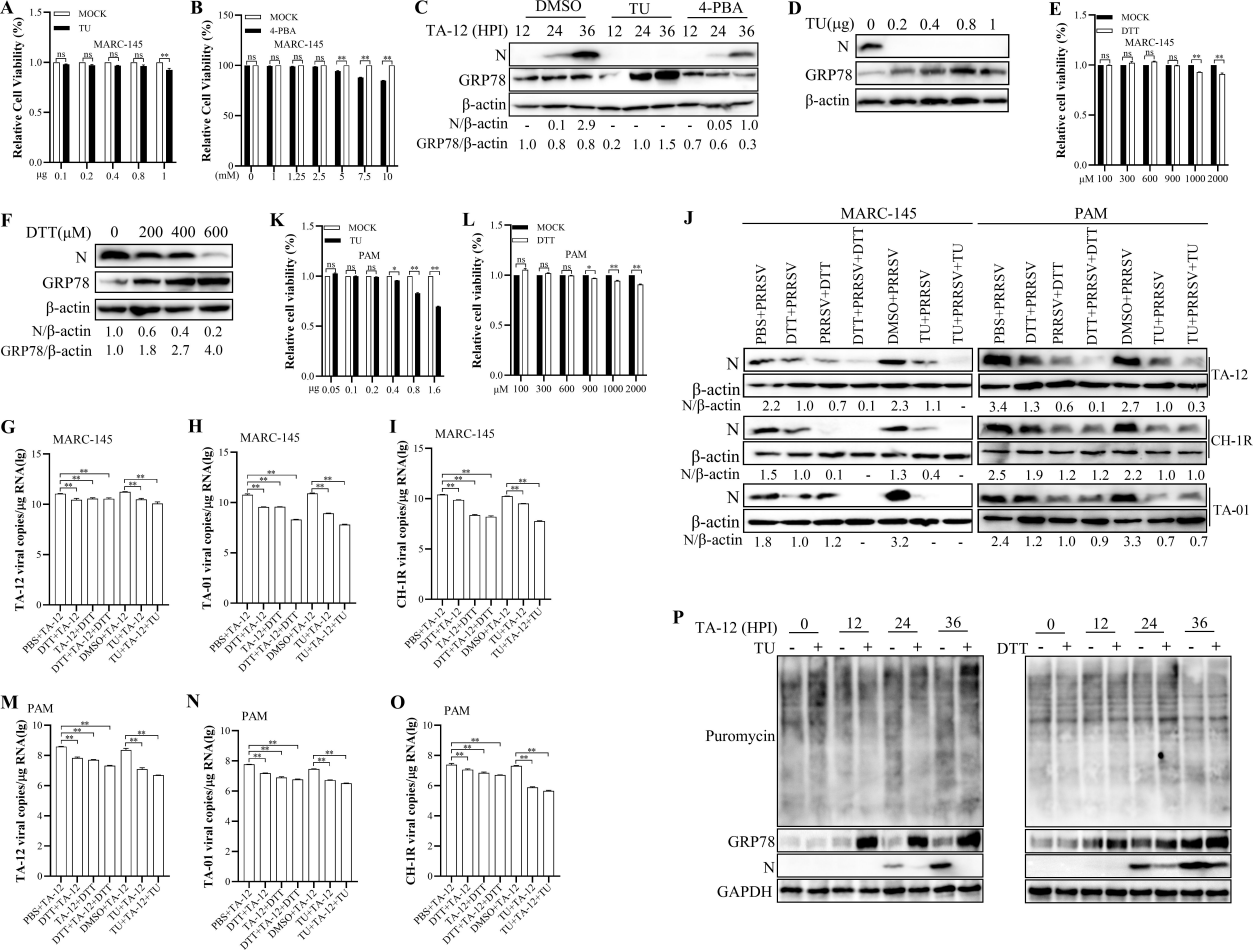
Pharmacological modulation of ER stress potently inhibits PRRSV replication. (**A and B**) Cytotoxicity assessment of ER stress modulators. MARC-145 cells in 96-well plates were treated with tunicamycin (TU) or 4-phenylbutyric acid (4-PBA). Cell viability was measured at 48 h post-treatment using the CCK-8 assay, with absorbance at 450 nm normalized to untreated controls (set as 100%). (**C**) Both ER stress induction and inhibition impair PRRSV replication. MARC-145 cells infected with the HP-PRRSV strain TA-12 were treated with TU or 4-PBA. Cell lysates were harvested at 12, 24, and 36 HPI and analyzed by Western blot for protein levels of the ER stress marker GRP78 and the viral N protein. (**D**) TU suppresses PRRSV replication in a dose-dependent manner. MARC-145 cells infected with TA-12 were treated with increasing concentrations of TU. Viral N protein levels were assessed by Western blot at 30 HPI. (**E**) Cytotoxicity of DTT on MARC-145 cells was evaluated by the CCK-8 assay. (**F**) The ER stress inducer DTT inhibits PRRSV replication. PRRSV-infected MARC-145 cells were treated with the indicated concentrations of DTT, and viral N protein levels were analyzed by Western blot at 30 HPI. (**G-J**) Broad-spectrum antiviral activity of TU and DTT in MARC-145 cells. Cells were treated with TU or DTT either before (prophylactic) or after (therapeutic) infection with diverse PRRSV strains (HP-PRRSV TA-12, NADC30-like TA-01, and recombinant TA-02). Viral replication was assessed at 30 HPI by qRT-PCR for genomic RNA (**G–I**) and by Western blot for N protein (**J**). (**K**) Cytotoxicity of TU on PAMs was evaluated by the CCK-8 assay. (**L**) Cytotoxicity of DTT on PAMs was evaluated by the CCK-8 assay. (**J, M–O**) Broad-spectrum antiviral activity of TU and DTT in PAMs. PAMs were treated with TU or DTT under prophylactic or therapeutic regimens and infected with the indicated PRRSV strains. Viral replication was assessed at 24 HPI by Western blot (**J**) and qRT-PCR (**M–O**). (**P**) Puromycin incorporation assay to monitor *de novo* protein synthesis. MARC-145 cells were infected with PRRSV strain TA-12 at a multiplicity of infection (MOI) of 0.1 and treated with DTT or TU as indicated. At 0, 12, 24, and 36 HPI, cells were pulse-labeled with 10 μM puromycin for 30 min to allow incorporation into nascent polypeptide chains. Newly synthesized proteins were then detected by immunoblotting using an anti-puromycin antibody. GAPDH or β-actin served as loading controls for qRT-PCR and Western blot, respectively. Data are presented as the mean ± SD from three independent experiments each performed with separate infections. Statistical significance was determined by an unpaired two-tailed Student’s *t*-test (*P < 0.05, **P < 0.01).

To define the role of ER stress in PRRSV replication, we first evaluated the cytotoxicity of pathway modulators on MARC-145 cells (Fig. 6A and B). Using non-cytotoxic concentrations, we found that both the ER stress inducer tunicamycin (TU) and the chemical chaperone 4-phenylbutyric acid (4-PBA) significantly inhibited PRRSV replication, with TU exhibiting a more potent, near-complete suppression (Fig. 6C). This indicates that productive viral replication depends on a precisely tuned, moderate ER stress environment and is disrupted by both excessive stress induction and attenuation of the stress response.

We further evaluated the antiviral potential of two established ER stress inducers, TU and dithiothreitol (DTT). Both compounds dose-dependently inhibited PRRSV replication (Fig. 6D through F). Given their shared mechanism of action, we assessed their individual and combined effects against diverse PRRSV strains. Treatment with TU or DTT alone significantly reduced the genomic RNA and protein levels of the HP-PRRSV strain TA-12, the NADC30-like strain TA-01, and a recombinant strain (TA-02) in MARC-145 cells (Fig. 6G through J). The combination of TU and DTT resulted in the most potent inhibitory effect. This broad-spectrum antiviral activity was confirmed in the natural host cell, porcine alveolar macrophages (PAMs), where both individual and combined treatments effectively suppressed all tested strains (Fig. 6K through O).

To exclude the possibility that the antiviral effect of TU and DTT stemmed from general inhibition of host translation, we performed a puromycin-incorporation protein synthesis assay. Puromycin is a structural analog of the aminoacyl-tRNA, which is incorporated into nascent polypeptide chains and can be detected via Western blot using anti-puromycin antibody (26, 27). The results revealed no reduction in puromycin incorporation, indicating that neither TU nor DTT impaired global host translation under the conditions used (Fig. 6P). Collectively, these results demonstrate that pharmacological agents that dysregulate ER homeostasis, particularly the inducers TU and DTT, possess potent and broad-spectrum antiviral activity against diverse PRRSV strains. This positions the targeted disruption of the virus-hijacked ER stress pathway as a promising strategy for developing novel interventions against PRRS.

## DISCUSSION

This study identifies the ER translocon component SSR4 as a critical host factor hijacked by PRRSV to promote viral replication. We demonstrate that Nsp2 interacts with SSR4, upregulates its expression, and induces a finely balanced ER stress response that favors viral replication.

The physical and functional interaction between Nsp2 and SSR4 represents a novel virus-host interface. While Nsp2 is a multifunctional protein involved in polyprotein processing and immune evasion (4–7), its direct binding to a core component of the ER translocon expands its functional repertoire. This interaction is not merely incidental; it is essential for viral fitness, as evidenced by the significant impairment of PRRSV replication upon SSR4 knockdown and its enhancement upon SSR4 overexpression (Fig. 5). Notably, this pro-viral role is specific to SSR4 within the TRAP complex. The selective upregulation of SSR4, but not other TRAP subunits (SSR1-3), upon infection suggests that PRRSV has evolved a mechanism to fine-tune the host ER translocon composition, specifically enriching for SSR4 to create a favorable niche for replication. However, the precise mechanism by which SSR4 promotes viral replication remains to be fully elucidated. SSR4 knockdown reduces viral protein and RNA levels (Fig. 2) and modulates ER stress signaling (Fig. 3 and 4), but whether SSR4 affects viral protein synthesis, replication complex formation, membrane remodeling, or virion assembly is unknown (28, 29). Additional studies are necessary for its proviral activity will be needed to address this question.

**Fig 7:**
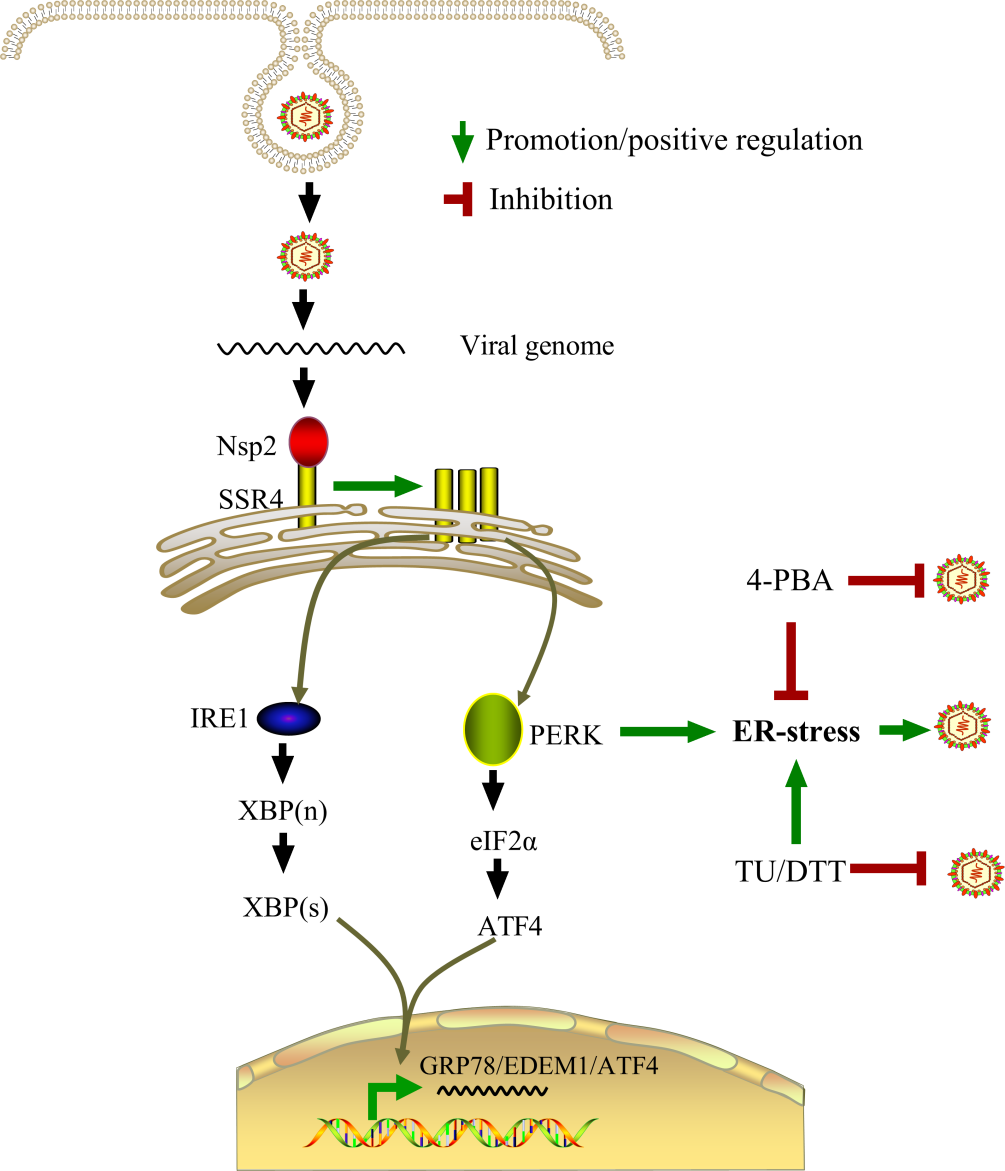
Model of the Nsp2-SSR4-ER stress axis in PRRSV infection. PRRSV entry initiates the expression of Nsp2. Nsp2 directly interacts with and upregulates SSR4. Upregulated SSR4, in turn, enhances ER stress signaling, which further amplifies Nsp2-driven ER stress activation, thereby establishing a feed-forward regulatory loop. This loop drives the full activation of UPR predominantly through the IRE1α and PERK pathways, leading to the upregulation of key ER chaperones and remodeling factors, such as GRP78, EDEM1, and ATF4. The consequent reprogramming of the ER facilitates the formation of a proviral niche that supports viral replication. Pharmacologically, both ER stress inducers (TU and DTT) and the inhibitor 4-PBA disrupt this finely balanced axis, each potently inhibiting PRRSV replication. These findings collectively highlight the Nsp2-SSR4-ER stress axis as a promising target for developing broad-spectrum antiviral strategies against PRRS.

SSR4 is a necessary amplifier for the full induction of ER stress markers (GRP78, ATF4, EDEM1, XBP1s) during infection (Fig. 3) and selectively modulates the PERK-eIF2α and IRE1α-XBP1 axes without engaging ATF6 (Fig. 4). This positions SSR4 as an active UPR regulator in the context of PRRSV infection (Fig. 7).

The ER stress response influences multiple cellular processes—including translation control, autophagy, and inflammatory signaling—that viruses can manipulate to their advantage (7, 30–32). In this study, it is intriguing to find that SSR4 is required for efficient viral replication, the pharmacologic data reveal that the relationship between ER stress and PRRSV is biphasic and finely balanced. The potent suppression of infection by both the ER stress inducer TU and the chemical chaperone 4-PBA indicates that PRRSV depends on a narrow, moderate window of ER stress activation (Fig. 6). Excessive, uncontrolled stress (induced by TU) is detrimental, likely triggering global translational shutoff and/or apoptosis. Conversely, suppressing the virus-induced stress response (via 4-PBA) also inhibits replication, confirming that the virus-beneficial pathways within the UPR are indispensable.

This paradox—PRRSV both induces ER stress and is inhibited by further induction—can be explained by quantitative and qualitative differences between virus-induced and pharmacologically induced stress. GRP78 levels were markedly lower in cells treated with 0.2 μg TU than in PRRSV-infected cells at 24–36 hpi (Fig. 3G vs Fig. 6D), suggesting that stress magnitude, not just presence, determines the outcome (15). PRRSV induces moderate ER stress that supports replication, while TU induces milder stress that nonetheless inhibits it, indicating that factors beyond amplitude—such as kinetics or UPR branch activation—are critical. PRRSV selectively activates PERK and IRE1α without engaging ATF6 (Fig. 4) (15), whereas TU activates all three branches simultaneously (17). This differential activation pattern may explain why virus-induced stress supports replication while pharmacologically induced stress inhibits it (15). However, quantitative comparison is complicated by differences in kinetics and cell heterogeneity. Defining the optimal ER stress window for PRRSV replication—including magnitude, duration, and UPR branch activation—will require more precise tools such as inducible expression systems or single-cell analyses.

The broad-spectrum antiviral activity of TU and DTT against multiple PRRSV strains, including HP-PRRSV and NADC30-like variants (Fig. 6), highlights the central and conserved nature of this virus-ER interplay. It further suggests that targeting the ER stress pathway, particularly the specific molecular interface we have identified, could be a viable therapeutic strategy (8, 33). However, it is important to acknowledge that these pharmacological agents have broader cellular effects beyond ER stress modulation. TU inhibits N-linked glycosylation (34), DTT is a reducing agent that affects disulfide bond formation (35), and 4-PBA has chaperone activity that may influence protein folding independently of ER stress pathways (36). While our puromycin incorporation assay suggests that TU and DTT do not globally inhibit host translation under the conditions used (Fig. 6P), other off-target effects cannot be excluded. Therefore, the observed antiviral effects may not be mediated solely through the Nsp2-SSR4-ER stress axis (37). More specific approaches—such as disrupting the Nsp2-SSR4 interaction directly using competitive peptides or small molecules, or genetically manipulating SSR4 expression *in vivo*—will be needed to validate this pathway as a bona fide therapeutic target (38). Our findings propose a model where small molecules or biologics designed to disrupt the Nsp2-SSR4 interaction or to precisely modulate its downstream signaling could act as effective antivirals.

Our findings demonstrate that Nsp2 stabilizes SSR4 at the post-translational level by prolonging its protein half-life without altering mRNA expression (Fig. 5). However, the precise mechanism by which Nsp2 mediates this stabilization remains incompletely defined. Several possibilities could account for this effect: direct stabilization through physical interaction with SSR4 (Fig. 1), interference with proteasomal degradation pathways, or indirect upregulation secondary to Nsp2-induced ER stress (39). While co-localization of Nsp2 with ER chaperones GRP78 and calnexin (Fig. 5) supports a direct role at the ER membrane, we cannot exclude the possibility that SSR4 upregulation occurs indirectly via ER stress signaling, as ER stress itself can modulate translocon-associated components (40, 41). Further studies are needed to distinguish between these potential mechanisms.

In conclusion, this study reveals that PRRSV hijacks SSR4 to modulate ER stress and promote replication. The Nsp2-SSR4 interface represents a potential target for broad-spectrum antiviral interventions against this economically devastating swine pathogen.

## MATERIALS AND METHODS

### Cells and virus

MARC-145 and 293T cells were obtained from the China Center for Type Culture Collection (CCTCC, Wuhan, China). Cells were cultured in Dulbecco’s modified Eagle’s medium (DMEM; Gibco) supplemented with 10% fetal bovine serum (FBS) at 37°C in a humidified 5% CO_2_ atmosphere. The highly pathogenic porcine reproductive and respiratory syndrome virus (HP-PRRSV) strain TA-12 (GenBank accession no. HQ416720) was isolated and preserved in our laboratory. NADC30-like strain TA-01 and recombinant strain (recombined by HP-PRRSV and NADC30-like) strain TA-02 were previously isolated and stored in our lab. PAMs were isolated from five healthy five-week-old crossbred weaned pigs (Landrace × Yorkshire) as previously described (42). The number of PAMs was adjusted to 2.5 × 10^6^/mL, and the aliquots were frozen in liquid nitrogen. Experiments involving PAMs were pooled and repeated three times to eliminate any differences among different pigs.

### Antibodies and reagents

The following primary antibodies were used: anti-β-actin (Proteintech, 66009-1-Ig), anti-Calnexin (Proteintech, 10427-2-AP), anti-SSR1 (Proteintech, 10583-1-AP), anti-SSR2 (Proteintech, 10278-1-AP), anti-SSR3 (Proteintech, 30851-1-AP), anti-SSR4 (Proteintech, 11655-2-AP), anti-ATF4 (Proteintech, 10835-1-AP), anti-EDEM1 (Proteintech, 26226-1-AP), anti-GRP78 (Beyotime, AF0171), anti-ATF6 (Proteintech, 24169-1-AP), anti-Phospho-eIF2 alpha (Cell Signaling Technology, #3398), anti-eIF2 alpha (Cell Signaling Technology, #5324), anti-Phospho-IRE1 (HUABIO, HA721980), anti-IRE1 (HUABIO, HA723225), anti-Puromycin (ABclonal, A21205), monoclonal anti-PRRSV N protein (generated in-house), and monoclonal anti-PRRSV Nsp2 (generated in-house). Key commercial reagents included tunicamycin (Beyotime, SC0393), DTT (Solarbio, 3483-12-3), 4-phenylbutyric acid (MCE, HY-A0281), MK-28 (AbMole, M56016), puromycin (MCE, HY-B1743), cycloheximide (MCE, HY-12320), BCA Protein Assay Kit (Thermo Fisher, 23225), Lipofectamine 3000 (Thermo Fisher, L3000015), Lipofectamine RNAiMAX (Thermo Fisher, 13778075), ReverTra Ace qPCR RT Kit (TOYOBO, NLQ-101), KOD qPCR SYBR Mix (TOYOBO, QKD-201T), tunicamycin (MedChemExpress, HY-A0098), RIPA lysis buffer (Beyotime, P0013C), DAPI staining solution (Beyotime, C1006), and polyethylenimine (PEI; Yeasen).

### Plasmid construction and transfection

The coding sequence of Nsp2 from the TA-12 strain was amplified by PCR and cloned into the pEGFP-C1 vector, as described previously (25). All plasmid constructs were verified by Sanger sequencing and are listed in Table 1. For transfection, HEK293T cells were transfected using PEI, whereas MARC-145 cells were transfected using Lipofectamine 3000.

**TABLE 1:** Primer sequences used for plasmid construction, quantitative PCR, and siRNA synthesis

Vector or purpose	Gene	Forward or sense (5’−3’)	Reverse or antisense (5’−3’)
pEGFP-C1	NSP2	AAGCTTCGAATTCTGCAATGGCCGGAAAGAGAGCAAGG	GGCCCGCGGTACCGTCGACTTACCCGAAGACCATTAACTTGC
qPCR	GAPDH	ACCCACTCTTCCACCTTCGACGCT	TGTTGTTGTAGCCAAATTCG
	SSR1	GAGGATGGGTTAGATGGAGAAAC	TTTCTGTATGGGTCTCTTACGC
	SSR2	TGGCTCTATTTGCTGTCACTC	GTACTGCAAGGTTAGGTCTCG
	SSRG	TGGTCGTGGTCATTGTTG	CCTGATGAAGCACTTATGGA
	SSRD	AGGAAGGCTCAGAGGAATA	GTAGTAGATCACAAGACCAATC
	GRP78	AGAAACTTCGGCGTGAGGTG	GAGTCGAGCCACCAACAAGA
	ATF-4	CAACCTCTTCCCCTTTCCC	CATCTGGCTTCCTATCTCCTTC
	EDEM1	TTGACTCTTGTTGATGCATTGGA	GCTTTCTGGAACTCGGATGAAT
	XBP1	AAACAGAGTAGCAGCGCAGACTGC	GGATCTCTAAGACTAGAGGCTTGGTG
	N	AGATCATCGCCCAACTAAAC	GACACAATTGCCGCTCACTA
siRNA	NC	UUCUCCGAACGUGUCACGUTT	ACGUGACACGUUCGGAGAATT
	SSR1		
	255	GCUUCACCAAGUGCAGAUATT	UAUCUGCACUUGGUGAAGCTT
	555	CCAAGAUGCAGUCUUCAAUTT	AUUGAAGACUGCAUCUUGGTT
	SSR4		
	110	CCACUUCUGACGCUGUCAUTT	AUGACAGCGUCAGAAGUGGTT
	183	CCAGAACAUGGCUCUCUAUTT	AUAGAGAGCCAUGUUCUGGTT

### siRNA transfection

siRNAs targeting specific genes were designed and synthesized by GenePharma (Shanghai, China). Transfections were performed in MARC-145 cells using Lipofectamine RNAiMAX according to the manufacturer’s protocol. The sequences of all siRNAs used in this study are provided in Table 1.

### Western blot analysis

Total protein was extracted with RIPA lysis buffer (50 mM Tris-HCl pH 7.4, 150 mM NaCl, 1 mM EDTA, 1% NP-40, 0.5% sodium deoxycholate, 0.1% SDS) supplemented with protease and phosphatase inhibitors. Protein concentrations were determined using the BCA Protein Assay Kit. Proteins were resolved by 12%–15% SDS-PAGE and transferred to PVDF membranes. After blocking with Rapid Blocking Buffer, membranes were incubated with primary antibodies overnight at 4°C, followed by incubation with horseradish peroxidase (HRP)-conjugated secondary antibodies for 1 h at room temperature. Protein bands were visualized with Clarity Western ECL substrate using an Azure 600 imaging system (Azure Biosystems).

### Co-immunoprecipitation (Co-IP) assay

Cell lysates were prepared using lysis buffer supplemented with cOmplete EDTA-free Protease Inhibitor Cocktail and incubated with rotation at 4°C for 30 min. After centrifugation at 500 × *g* for 10 min, 500 µg of supernatant protein was subjected to immunoprecipitation with anti-GFP antibody-bound Protein A/G MagBeads overnight at 4°C. The immunoprecipitants were subsequently washed five times with lysis buffer, eluted in 5× loading buffer, and analyzed by SDS-PAGE followed by Western blot.

### Confocal microscopy

Cells were fixed with 4% paraformaldehyde for 15 min and permeabilized with 0.1% Triton X-100 in PBS. After blocking with 1% bovine serum albumin for 1 h, cells were incubated with primary antibodies for 1 h at room temperature, followed by incubation with fluorescently labeled secondary antibodies (FITC- or Cy3-conjugated). Nuclei were counterstained with DAPI. Images were acquired using a fluorescence microscope (Andor Dragonfly, Oxford Instruments).

### qRT-PCR analysis

Total RNA was isolated using TRIzol reagent and reverse transcribed with the ReverTra Ace qPCR RT Kit (TOYOBO). Quantitative real-time PCR was performed using KOD SYBR qPCR Mix (TOYOBO) on a LightCycler 96 system (Roche). All primer sequences are provided in Table 1. Gene expression levels were normalized to GAPDH and calculated using the 2^–ΔΔCt^ method.

### Puromycin incorporation protein synthesis assay

To measure global protein synthesis rates, a puromycin incorporation assay was performed. Puromycin is a structural analog of aminoacyl-tRNA, which is incorporated into nascent polypeptide chains and can be detected via Western blot using an anti-puromycin antibody, thereby providing a measure of mRNA translation in cells. MARC-145 cells infected with TA-12 were treated with TU and DTT, followed by 30 min of puromycin treatment at a final concentration of 10 µM prior to sample collection at designated time points. This concentration and duration of the treatment were optimized in preliminary experiments to ensure robust signal detection without significant toxicity.

### Virus titer assay

Viral titers were determined by endpoint dilution assay in MARC-145 cells. Briefly, serial 10-fold dilutions of virus samples were prepared in culture medium and inoculated into confluent monolayers of MARC-145 cells in 96-well plates. Viral titers were calculated using the Reed-Muench method and expressed as 50% tissue culture infectious dose (TCID50) per milliliter.

### XBP1 mRNA splicing assay

Total RNA was isolated from cultured cells using TRIzol reagent according to the manufacturer’s protocol. RNA concentration was quantified. cDNA was synthesized using the ReverTra Ace qPCR RT Kit. The XBP1 transcript was amplified by PCR with the primers listed in Table 1. To distinguish between the spliced (XBP1s) and unspliced (XBP1u) isoforms, PCR products were digested with PstI-HF restriction enzyme (NEB, #R3140L). Since XBP1 splicing removes a PstI restriction site, the inactive form XBP1(u) is cleaved into two small fragments, while XBP1s remains unchanged and analyzed by electrophoresis on 1.5% agarose gels.

### Protein stability assay

The half-life of the SSR4 protein was assessed using a cycloheximide (CHX) chase assay. Cells were seeded in six-well plates and grown to approximately 70%–80% confluence. Twenty-four hours after transfection with the Nsp2 expression plasmid, cells were treated with CHX at a final concentration of 5 μM to inhibit *de novo* protein synthesis. Whole-cell lysates were harvested at the indicated time points (0, 3, 5, and 8 h) post-CHX treatment, and SSR4 protein levels were analyzed by immunoblotting.

### Cell viability

The cytotoxicity of ER stress inducer tunicamycin (TU), ER stress inhibitor 4-phenylbutyric acid (4-PBA), and 1,4-dithiothreitol (DTT) was evaluated by the CCK-8 assay in accordance with the manufacturer’s instructions. MARC-145 cells or PAMs were grown in each well of 96-well plates to form monolayers. TU, 4-PBA, DTT, or MK-28 was added to the wells at specific concentrations, and the cells were further cultured for 48 h, following which the CCK-8 reagent was added to each well. After incubation for 2 h at 37°C, cell viability was evaluated by measuring absorbance at 450 nm. The optical density of wells containing untreated control cells was defined as indicating 100% viability.

### Pharmacological treatments and experimental regimens

For the evaluation of prophylactic and therapeutic potential, MARC-145 cells were treated with tunicamycin (TU) or dithiothreitol (DTT) according to three regimens: (i) a prophylactic regimen (TU/DTT + Virus), where compounds were added 2 h prior to viral inoculation; (ii) a therapeutic regimen (Virus + TU/DTT), where compounds were added 2 h post-inoculation; and (iii) a combined prophylactic-therapeutic regimen (TU/DTT + Virus + TU/DTT), where compounds were administered both before and after inoculation.

### Statistical analysis

Statistical analyses were performed using SPSS 20.0 (SPSS Inc.). Data were analyzed by one-way ANOVA or Student’s *t*-test, as appropriate. Results are presented as mean ± standard deviation (SD) from a minimum of three independent experiments. Differences with *P* < 0.05 were considered statistically significant.

## Data Availability

All data supporting the findings of this study are included in the article. Additional raw data are available from the corresponding author upon reasonable request.

## References

[1] Neumann EJ, Kliebenstein JB, Johnson CD, Mabry JW, Bush EJ, Seitzinger AH, Green AL, Zimmerman JJ. 2005. Assessment of the economic impact of porcine reproductive and respiratory syndrome on swine production in the United States. J Am Vet Med Assoc 227:385–392. doi:10.2460/javma.2005.227.385 16121604

[2] Rossow KD. 1998. Porcine reproductive and respiratory syndrome. Vet Pathol 35:1–20. doi:10.1177/030098589803500101 9545131

[3] Lunney JK, Benfield DA, Rowland RRR. 2010. Porcine reproductive and respiratory syndrome virus: an update on an emerging and re-emerging viral disease of swine. Virus Res 154:1–6. doi:10.1016/j.virusres.2010.10.009 20951175 PMC7172856

[4] Zhang H, Sha H, Qin L, Wang N, Kong W, Huang L, Zhao M. 2022. Research progress in porcine reproductive and respiratory syndrome virus–host protein interactions. Animals (Basel) 12:1381. doi:10.3390/ani12111381 35681845 PMC9179581

[5] Li J, Zhang J, Sun P, Wang J, Li G, Cui Z, Li D, Yuan H, Wang T, Li K, Bai X, Zhao Z, Cao Y, Ma X, Li P, Fu Y, Bao H, Liu Z, Xiao S, Wang X, Lu Z. 2024. Porcine reproductive and respiratory syndrome virus nonstructural protein 2 promotes the autophagic degradation of adaptor protein SH3KBP1 to antagonize host innate immune responses by enhancing K63-linked polyubiquitination of RIG-I. PLoS Pathog 20:e1012670. doi:10.1371/journal.ppat.1012670 39466846 PMC11560026

[6] Cao S, Liu J, Ding G, Shao Q, Wang B, Li Y, Feng J, Zhao Y, Liu S, Xiao Y. 2020. The tail domain of PRRSV NSP2 plays a key role in aggrephagy by interacting with 14-3-3ε. Vet Res 51:104. doi:10.1186/s13567-020-00816-7 32811532 PMC7433210

[7] Diao F, Jiang C, Sun Y, Gao Y, Bai J, Nauwynck H, Wang X, Yang Y, Jiang P, Liu X. 2023. Porcine reproductive and respiratory syndrome virus infection triggers autophagy via ER stress-induced calcium signaling to facilitate virus replication. PLoS Pathog 19:e1011295. doi:10.1371/journal.ppat.1011295 36972295 PMC10079224

[8] Hetz C, Papa FR. 2018. The unfolded protein response and cell fate control. Mol Cell 69:169–181. doi:10.1016/j.molcel.2017.06.017 29107536

[9] Fung TS, Huang M, Liu DX. 2014. Coronavirus-induced ER stress response and its involvement in regulation of coronavirus-host interactions. Virus Res 194:110–123. doi:10.1016/j.virusres.2014.09.016 25304691 PMC7114476

[10] Ron D, Walter P. 2007. Signal integration in the endoplasmic reticulum unfolded protein response. Nat Rev Mol Cell Biol 8:519–529. doi:10.1038/nrm2199 17565364

[11] Credle JJ, Finer-Moore JS, Papa FR, Stroud RM, Walter P. 2005. On the mechanism of sensing unfolded protein in the endoplasmic reticulum. Proc Natl Acad Sci USA 102:18773–18784. doi:10.1073/pnas.0509487102 16365312 PMC1316886

[12] Karagöz GE, Acosta-Alvear D, Nguyen HT, Lee CP, Chu F, Walter P. 2017. An unfolded protein-induced conformational switch activates mammalian IRE1. eLife 6:e30700. doi:10.7554/eLife.30700 28971800 PMC5699868

[13] Pincus D, Chevalier MW, Aragón T, van Anken E, Vidal SE, El-Samad H, Walter P. 2010. BiP binding to the ER-stress sensor Ire1 tunes the homeostatic behavior of the unfolded protein response. PLoS Biol 8:e1000415. doi:10.1371/journal.pbio.1000415 20625545 PMC2897766

[14] Xue M, Fu F, Ma Y, Zhang X, Li L, Feng L, Liu P. 2018. The PERK arm of the unfolded protein response negatively regulates transmissible gastroenteritis virus replication by suppressing protein translation and promoting type I interferon production. J Virol 92:e00431-18. doi:10.1128/JVI.00431-18 29769338 PMC6052291

[15] Chen Q, Men Y, Wang D, Xu D, Liu S, Xiao S, Fang L. 2020. Porcine reproductive and respiratory syndrome virus infection induces endoplasmic reticulum stress, facilitates virus replication, and contributes to autophagy and apoptosis. Sci Rep 10:13131. doi:10.1038/s41598-020-69959-z 32753633 PMC7403369

[16] Huo Y, Fan L, Yin S, Dong Y, Guo X, Yang H, Hu H. 2013. Involvement of unfolded protein response, p53 and Akt in modulation of porcine reproductive and respiratory syndrome virus-mediated JNK activation. Virology (Auckl) 444:233–240. doi:10.1016/j.virol.2013.06.015 23850458

[17] Catanzaro N, Meng XJ. 2020. Induction of the unfolded protein response (UPR) suppresses porcine reproductive and respiratory syndrome virus (PRRSV) replication. Virus Res 276:197820. doi:10.1016/j.virusres.2019.197820 31743697

[18] Chen W-Y, Schniztlein WM, Calzada-Nova G, Zuckermann FA. 2018. Genotype 2 strains of porcine reproductive and respiratory syndrome virus dysregulate alveolar macrophage cytokine production via the unfolded protein response. J Virol 92:e01251-17. doi:10.1128/JVI.01251-17 PMC5752938 29070690

[19] Zhu Z, Liu P, Yuan L, Lian Z, Hu D, Yao X, Li X. 2021. Induction of UPR promotes interferon response to inhibit PRRSV replication via PKR and NF-κB pathway. Front Microbiol 12:757690. doi:10.3389/fmicb.2021.757690 34712218 PMC8547762

[20] Gao P, Chai Y, Song J, Liu T, Chen P, Zhou L, Ge X, Guo X, Han J, Yang H. 2019. Reprogramming the unfolded protein response for replication by porcine reproductive and respiratory syndrome virus. PLoS Pathog 15:e1008169. doi:10.1371/journal.ppat.1008169 31738790 PMC6932825

[21] Phoomak C, Cui W, Hayman TJ, Yu SH, Zhao P, Wells L, Steet R, Contessa JN. 2021. The translocon-associated protein (TRAP) complex regulates quality control of N-linked glycosylation during ER stress. Sci Adv 7:eabc6364. doi:10.1126/sciadv.abc6364 33523898 PMC7810369

[22] Hartmann E, Görlich D, Kostka S, Otto A, Kraft R, Knespel S, Bürger E, Rapoport TA, Prehn S. 1993. A tetrameric complex of membrane proteins in the endoplasmic reticulum. Eur J Biochem 214:375–381. doi:10.1111/j.1432-1033.1993.tb17933.x 7916687

[23] Jaskolowski M, Jomaa A, Gamerdinger M, Shrestha S, Leibundgut M, Deuerling E, Ban N. 2023. Molecular basis of the TRAP complex function in ER protein biogenesis. Nat Struct Mol Biol 30:770–777. doi:10.1038/s41594-023-00990-0 37170030 PMC10279528

[24] Lakkaraju AKK, Thankappan R, Mary C, Garrison JL, Taunton J, Strub K. 2012. Efficient secretion of small proteins in mammalian cells relies on Sec62-dependent posttranslational translocation. MBoC 23:2712–2722. doi:10.1091/mbc.e12-03-0228 22648169 PMC3395660

[25] Xiao Y, Wu W, Gao J, Smith N, Burkard C, Xia D, Zhang M, Wang C, Archibald A, Digard P, Zhou EM, Hiscox JA. 2016. Characterization of the interactome of the porcine reproductive and respiratory syndrome virus nonstructural protein 2 reveals the hyper variable region as a binding platform for association with 14-3-3 proteins. J Proteome Res 15:1388–1401. doi:10.1021/acs.jproteome.5b00396 26709850

[26] Hadidi K, Steinbuch KB, Dozier LE, Patrick GN, Tor Y. 2023. Inherently emissive puromycin analogues for live cell labelling. Angew Chem Int Ed 62:23. doi:10.1002/anie.202216784 PMC10213139 36973168

[27] Schmidt EK, Clavarino G, Ceppi M, Pierre P. 2009. SUnSET, a nonradioactive method to monitor protein synthesis. Nat Methods 6:275–277. doi:10.1038/nmeth.1314 19305406

[28] Reid DW, Chen Q, Tay AS-L, Shenolikar S, Nicchitta CV. 2014. The unfolded protein response triggers selective mRNA release from the endoplasmic reticulum. Cell 158:1362–1374. doi:10.1016/j.cell.2014.08.012 25215492 PMC4163055

[29] Zhang L, Wang A. 2020. Virus-induced ER stress and the unfolded protein response. Front Plant Sci 11:820. doi:10.3389/fpls.2012.00293 23293645 PMC3531707

[30] Jiao P, Fan W, Ma X, Lin R, Zhao Y, Li Y, Zhang H, Jia X, Bi Y, Feng X, Li M, Liu W, Zhang K, Sun L. 2023. SARS-CoV-2 nonstructural protein 6 triggers endoplasmic reticulum stress-induced autophagy to degrade STING1. Autophagy 19:3113–3131. doi:10.1080/15548627.2023.2238579 37482689 PMC10621274

[31] Zhang L-K, Wang B, Xin Q, Shang W, Shen S, Xiao G, Deng F, Wang H, Hu Z, Wang M. 2019. Quantitative proteomic analysis reveals unfolded-protein response involved in severe fever with thrombocytopenia syndrome virus infection. J Virol 93:e00308–19. doi:10.1128/JVI.00308-19 30842332 PMC6498065

[32] Ma Y, Wang C, Xue M, Fu F, Zhang X, Li L, Yin L, Xu W, Feng L, Liu P. 2018. The coronavirus transmissible gastroenteritis virus evades the type i interferon response through IRE1α-mediated manipulation of the MicroRNA miR-30a-5p/SOCS1/3 Axis. J Virol 92:e00728-18. doi:10.1128/JVI.00728-18 30185587 PMC6206482

[33] Almanza A, Carlesso A, Chintha C, Creedican S, Doultsinos D, Leuzzi B, Luís A, McCarthy N, Montibeller L, More S, Papaioannou A, Püschel F, Sassano ML, Skoko J, Agostinis P, de Belleroche J, Eriksson LA, Fulda S, Gorman AM, Healy S, Kozlov A, Muñoz-Pinedo C, Rehm M, Chevet E, Samali A. 2019. Endoplasmic reticulum stress signalling - from basic mechanisms to clinical applications. FEBS J 286:241–278. doi:10.1111/febs.14608 30027602 PMC7379631

[34] Elbein AD. 1987. Inhibitors of the biosynthesis and processing of N-linked oligosaccharide chains. Annu Rev Biochem 56:497–534. doi:10.1146/annurev.bi.56.070187.002433 3304143

[35] Braakman I, Helenius J, Helenius A. 1992. Manipulating disulfide bond formation and protein folding in the endoplasmic reticulum. EMBO J 11:1717–1722. doi:10.1002/j.1460-2075.1992.tb05223.x 1582407 PMC556629

[36] Kolb PS, Ayaub EA, Zhou W, Yum V, Dickhout JG, Ask K. 2015. The therapeutic effects of 4-phenylbutyric acid in maintaining proteostasis. Int J Biochem Cell Biol 61:45–52. doi:10.1016/j.biocel.2015.01.015 25660369

[37] Dawood AA, Altobje MA. 2020. Inhibition of N-linked Glycosylation by Tunicamycin May Contribute to The Treatment of SARS-CoV-2. Microb Pathog 149:104586. doi:10.1016/j.micpath.2020.104586 33091582 PMC7573633

[38] Scott DE, Bayly AR, Abell C, Skidmore J. 2016. Small molecules, big targets: drug discovery faces the protein-protein interaction challenge. Nat Rev Drug Discov 15:533–550. doi:10.1038/nrd.2016.29 27050677

[39] Komander D, Rape M. 2012. The ubiquitin code. Annu Rev Biochem 81:203–229. doi:10.1146/annurev-biochem-060310-170328 22524316

[40] Fang Y, Fang L, Wang Y, Lei Y, Luo R, Wang D, Chen H, Xiao S. 2012. Porcine reproductive and respiratory syndrome virus nonstructural protein 2 contributes to NF-κB activation. Virol J 9:83. doi:10.1186/1743-422X-9-83 22546080 PMC3443020

[41] Sogawa A, Komori R, Yanagitani K, Ohfurudono M, Tsuru A, Kadoi K, Kimata Y, Yoshida H, Kohno K. 2023. Signal sequence-triage is activated by translocon obstruction sensed by an ER stress sensor IRE1α. Cell Struct Funct 48:211–221. doi:10.1247/csf.23072 37766570 PMC11496779

[42] Ding G, Liu J, Shao Q, Wang B, Feng J, Li Y, Li L, Cao S, Cong F, Zhao Y, Liu S, Xiao Y. 2020. Porcine reproductive and respiratory syndrome virus structural protein GP3 regulates claudin 4 To facilitate the early stages of infection. J Virol 94:e00124-20. doi:10.1128/JVI.00124-20 32759320 PMC7527063

